# Empathic behavior in nursing and medicine students

**DOI:** 10.15649/cuidarte.4007

**Published:** 2024-12-10

**Authors:** Yolima Pertuz-Meza, Luz Angela Reyes-Ríos, José Gamarra-Moncayo, Fernando Reyes-Reyes, Alejandro Reyes-Reyes, Víctor Díaz-Narváez

**Affiliations:** 1 Universidad Cooperativa de Colombia. Santa Marta, Colombia. E-mail: yolima.pertuz@campusucc.edu.co Universidad Cooperativa de Colombia Universidad Cooperativa de Colombia Santa Marta Colombia yolima.pertuz@campusucc.edu.co; 2 Universidad Cooperativa de Colombia. Santa Marta, Colombia. E-mail: luz.reyes@campusucc.edu.co Universidad Cooperativa de Colombia Universidad Cooperativa de Colombia Santa Marta Colombia luz.reyes@campusucc.edu.co; 3 Universidad Católica Santo Toribio de Mogrovejo. Chiclayo, Perú. E-mail: gamarramoncayoj@gmail.com Universidad Católica Santo Toribio de Mogrovejo Universidad Católica Santo Toribio de Mogrovejo Chiclayo Peru gamarramoncayoj@gmail.com; 4 Universidad del Desarrollo. Concepción, Chile. E-mail: freyes@udd.cl Universidad del Desarrollo Universidad del Desarrollo Concepción Chile freyes@udd.cl; 5 Universidad Santo Tomás. Concepción, Chile. E-mail: areyesr@santotomas.cl Universidad Santo Tomás Universidad Santo Tomás Concepción Chile areyesr@santotomas.cl; 6 Universidad Andres Bello. Santiago, Chile. E-mail: victor.diaz@unab.cl vicpadina@gmail.com Universidad Andrés Bello Universidad Andres Bello Santiago Chile victor.diaz@unab.cl

**Keywords:** Empathy, Medicine, Nursing, Student, Psychometry, Empatía, Medicina, Enfermería, Estudiante, Psicometría, Empatia, Medicina, Enfermagem, Estudante, Psicometria

## Abstract

**Introduction:**

Empathy is an attribute that contributes to humane care of patients and increases the likelihood of successful treatment.

**Objective:**

To measure the levels of empathy and its dimensions, and diagnose empathic behavior in medicine and nursing students.

**Materials and Methods:**

This was a non-experimental, cross-sectional study in which Colombian nursing and medicine students were evaluated using the Jefferson Scale of Empathy (JSE). A factor analysis, an analysis of invariance, and a two-way analysis of variance (ANOVA) were used, and the data were compared to specific cut-off points for each program. Effect size and power of the tests were estimated for the comparisons performed.

**Results:**

The theoretical model for the construct ‘empathy’ fitted the observed data, and the invariance of the model between groups was confirmed. Differences were observed between nursing and medical students, as well as between sexes, with results favoring the women group.

**Discussion:**

The differences found between students could be partly due to the curricular differences of the programs and the students' interests, whereas those related to sex may be due to characteristics observed in Latin American population. The identified empathy deficiencies allowed for the development of a diagnosis for nursing and medicine students.

**Conclusion:**

Based on the findings, it is possible to diagnose the empathy levels of students of both programs by identifying the deficiencies observed in the dimensions of empathy.

## Resumen.

**Introducción:**

La empatía es un atributo que contribuye a una atención humanizada de los pacientes y aumenta la probabilidad de éxito del tratamiento.

**Objetivo:**

Medir los niveles de empatía y sus dimensiones y diagnosticar el comportamiento empático de los estudiantes de medicina y enfermería.

**Materiales y Métodos:**

Estudio no experimental de corte transversal en el que se evaluó a estudiantes colombianos de enfermería y medicina mediante la Escala Jefferson de Empatía (EJE). Se utilizó un análisis factorial, un análisis de invarianza y un análisis de varianza (ANOVA) bifactorial, y los datos se compararon con puntos de corte específicos para cada uno de los programas. Se estimó el tamaño del efecto y la potencia de las pruebas de las comparaciones realizadas.

**Resultados:**

El modelo teórico para el constructo “empatía” se ajusta con los datos observados y se confirmó la invariabilidad del modelo entre los grupos. Se observaron diferencias entre los estudiantes de enfermería y los de medicina, así como entre sexos, con resultados favorables en el grupo de mujeres.

**Discusión:**

Las diferencias encontradas entre estudiantes podrían deberse a las diferencias curriculares de los programas y a los intereses de los estudiantes, mientras que las relacionadas con el sexo podrían deberse a características observadas en la población latinoamericana. Las deficiencias de empatía detectadas permitieron dar un diagnóstico para los estudiantes de los programas de enfermería y medicina.

**Conclusiones:**

Con base en los hallazgos, es posible proporcionar un diagnóstico del nivel de empatía de los estudiantes de ambos programas identificando las deficiencias observadas en las dimensiones de la empatía.

## Resumo.

**Introdução:**

A empatia é um atributo que contribui para o atendimento humanizado ao paciente e aumenta a probabilidade de sucesso do tratamento.

**Objetivo:**

Medir os níveis de empatia e suas dimensões e obter um diagnóstico do comportamento empático dos estudantes de medicina e enfermagem estudados.

**Materiais e Métodos:**

Estudo não experimental, com desenho de corte transversal. Foram examinados estudantes de enfermagem e de medicina da Colômbia. Foi aplicada a Escala Jefferson. Foram utilizadas a análise fatorial, a análise de invariância e a análise de variância de dois fatores. Os dados de empatia foram comparados com pontos de corte específicos para cada um deles. O tamanho do efeito e o poder dos testes das comparações feitas foram estimados.

**Resultados:**

O modelo teórico do construto de “empatia” se ajusta aos dados observados e a invariância do modelo entre os grupos foi confirmada. Foram observadas diferenças entre estudantes de enfermagem e de medicina, bem como entre os sexos, com resultados favoráveis no grupo feminino.

**Discussão:**

As diferenças encontradas entre os estudantes podem ser devidas às diferenças curriculares dos programas e aos interesses dos estudantes, enquanto as relacionadas ao gênero podem ser devidas às características observadas na população latino-americana. As deficiências de empatia detectadas permitiram que fosse feito um diagnóstico para os estudantes dos programas de enfermagem e medicina.

**Conclusões:**

Com base nos resultados, é possível fornecer um diagnóstico do nível de empatia dos estudantes em ambos os programas, identificando as deficiências observadas nas dimensões da empatia.

## Introduction

Empathy is an important human attribute in patient care that nursing and medical staff should have^1^^, ^^2^, as it allows the intersubjectivity between nurses, doctors and patients to be dynamically and unambiguously structured^3^^, ^^4^. Empathy comprises two components^1^^, ^^2^ (cognitive and emotional) and three dimensions^1^. The cognitive component, which includes two dimensions, helps healthcare workers gain insight into the patient's mind and understand their perspective. The emotional component, which has one dimension, allows healthcare workers to understand the patient's suffering while regulating this understanding to prevent emotional contagion^5^.

Latin American studies on empathy have proven the large variability of empathic behaviors^6^^, ^^7^^)^ influenced by factors such as sex^8^^, ^^10^ and the empathy decline process^11^. This variability has led to the hypothesis that no general patterns of empathic behavior exist among nursing and medicine students in Latin America. Instead, empathic behavior appears to be represented by different patterns. This situation suggests that the interventions aimed at increasing empathy levels should be preceded by a detailed diagnosis of empathy tailored to nursing and medicine students.

Empathy can be studied using several instruments that have been well-documented^12^. The Jefferson Scale of Physician Empathy is the most widely used scale in Latin America^13^^, ^^15^. This scale includes three dimensions: "Perspective-taking" (PT) and "Standing in the patient's shoes" (SPS), which comprise the cognitive component, and “Compassionate care” (CC), which represents the emotional component.

Data on empathy should undergo psychometric study with two essential objectives: to verify that the three-dimensional model fits and to determine that this model fits each group of interest within the studied population to compare the results of empathy and each of its dimensions^15^^, ^^17^.

Diagnosing empathy is a process of analysis and synthesis that involves examining each dimension individually and comparing the values obtained with cut-off points for medicine^16^ and nursing^17^^)^ in Latin America. The results obtained for each dimension are studied as a whole, and conclusions are drawn about the robustness or deficiency of empathy that can be observed in the populations studied.

Consequently, this study aims to measure the levels of empathy and its dimensions and reach a diagnosis of empathic behavior among university students in medicine and nursing.

## Materials and Methods

### Study design

This study used a non-experimental, descriptive, and cross-sectional design. The independent variables were the programs under study (nursing and medicine) and sex, while the dependent variables were empathy and its dimensions.

### Participants

The study population consisted of students from the nursing (N = 290) and medicine (N = 535) programs atthe School ofHealth Sciences ofthe Universidad Cooperativa de Santa Marta, Colombia (2023). The sample included all students enrolled in the courses of each program who voluntarily completed the JSE. Therefore, the sampling method can be regarded as a non-probability and convenience sampling.

### Inclusion criterion

All students enrolled in these university programs who voluntarily completed the JSE on the day it was administered were included in the study. Data were collected by trained faculty personnel, and both the instrument and the informed consent form were completed in person and in written form. Each student received a copy of the informed consent form.

### Instrument

Empathy was assessed using the Jefferson Scale of Empathy's Health Professions Students' version (JSE HPS-Version)^18^. It is a 20-item Likert-type scale, with responses ranging from 1 to 7, with minimum values of 20 points (low empathy levels) and maximum values of 140 points (high empathy levels) ^15^^, ^^17^.

### Operationalization

Empathy is measured by quantifying the instrument score (20-140 points). The dimensions are scored as follows: Compassionate care (CC) with 8 items and up to 56 points, perspective taking (PT) with 10 items and up to 70 points, and standing in the patient's shoes (SPS) with 2 items and up to 14 points.

### Procedure

Before using the instrument, a content validity process was conducted through expert judgement. The instrument was also pilot-tested on 20 students, male and female, selected from all courses within each cohort, before it was used on a large scale^19^.

### Data analysis: Psychometric properties

A confirmatory factor analysis (CFA) was conducted using maximum likelihood with robust standard errors (MLR)^20^, as the items had more than five response categories^21^. The fit indices used were the following: root mean square error of approximation (RMSEA < .08), standardized root mean square residual (SRMR < .08), comparative fit index (CFI > .95), and Tucker-Lewis index (TLI > .95) ^22^^, ^^24^. The internal consistency of the scale was assessed using the Omega coefficient^25^, with a value greater than .70 considered adequate^26^.

The factorial invariance of the scale in function of the programs and sex was evaluated using hierarchical invariance models: configural invariance, metric invariance, scalar invariance, and strict invariance. To compare differences in the sequence of models, differences in RMSEA (ARMSEA) were used, and differences of less than <.015 indicated model invariance^27^.

Descriptive analyses were conducted to assess univariate normality (skewness and kurtosis) and multivariate normality (Mardia's test), followed by a CFA using the MLR estimator. Fit indices *CFI > .*90, *TLI > .*90, *RMSEA < .*08 *y SRMR <* .05 indicated proper model fit^28^. The Omega coefficient was used to assess reliability, with values above .70 considered adequate^29^^, ^^30^. Descriptive statistics, including mean and standard deviation, were calculated for empathy and each of its dimensions. A two-way analysis of variance (ANOVA) was used to compare empathy levels and empathy by its dimensions, with program (nursing and medicine) and sex (male and female) as factor variables, and included an interaction estimate between factor levels. Effect size (n2) and test power (1-P) were calculated for each comparison^31^, as well as the adjusted coefficient of determination R2. The analyses were performed using the R and its RStudio interface, running the packages Lavaan (v0.6- 17), Psych (v2.4.1), semTools (v0.5-6), and MVN (v5.9). Statistical package SPSS (v25.0) was used for the ANOVA. The significance level for hypothesis testing was set at a < 0.05, with a test power of 1-p > 0.80. The data are available in the OSF repository^32^.

### Ethical considerations

This research study was approved by the Institutional Ethics Committee of Andres Bello University in Santiago, Chile, Resolution No. 020/2022.

## Results

The nursing students sample comprised 237 participants (81.70% of the total; 20.70% male and 79.30% female), while the medical student sample comprised 436 participants (81.50% ofthe total; 28.00% male and 72.00% female).

The mean age and its standard deviation were estimated by program and sex: medical students, 20.50 ± 3.93; nursing students, 20.32 ± 3.93; male participants, 20.46 ± 3.14; and female participants: 20.43 ± 3.11.

Table 1 shows that no item exceeded the cut-off values for skewness (+/- 2) and kurtosis (+/- 7), allowing univariate normality to be assumed. The only relatively low mean value was observed in item 18.

**Table 1:** Univariate descriptive statistics for empathy scale items

Items	M±SD	SD	Asymmetry	Kurtosis
1	4.23	2.12	-0.08	-1.36
2	5.80	1.68	-1.47	1.20
3	4.14	1.69	0.04	-0.75
4	5.74	1.70	-1.42	1.08
5	5.18	1.74	-0.76	-0.36
6	3.92	1.71	0.15	-0.66
7	5.11	2.11	-0.73	-0.90
8	4.64	1.99	-0.36	1.14
9	5.51	1.66	1.02	0.16
10	5.23	1.63	-0.88	0.13
11	4.78	1.91	-0.46	-0.93
12	4.86	2.08	-0.55	-1.10
13	5.48	1.70	-0.96	0.00
14	5.14	1.90	-0.67	-0.77
15	5.13	1.78	-0.72	-0.42
16	5.49	1.59	-0.95	0.23
17	4.96	1.79	-0.59	-0.58
18	3.34	1.93	0.41	-0.96
19	4.77	1.94	-0.44	-0.99
20	5.90	1.58	-1.45	1.18

*Note: M = Mean; SD = Standard Deviation.*

Multivariate normality was evaluated using Mardia's test, which showed that the data did not meet this condition (p < 0.001). Therefore, using the MLR estimator for the CFA was convenient.

The original correlated three-factor model was initially tested using the MLR estimator and showed an adequate fit. However, upon reviewing the factor loadings, item 18 had a substantially lower loading (0.05) than the others, so it was decided to respecify the model by leaving out this item. The revised model showed slight improvements in terms of CFI, TLI, and SRMR (Table 2).

**Table 2:** Dependent variables of the study by patient's treatment specialty

Models	X2 (df)	CFI	TLI	RMSEA [CI 90%]	SRMR
1). Three correlated factors	378.19 (167)	0.92	0.91	0.04 [.04 - 0.05]	0.06
2). Three correlated factors (leaving out item 18)	326.15 (149)	0.93	0.92	0.04 [.004 - 0.05]	0.05

**Figure 1:**
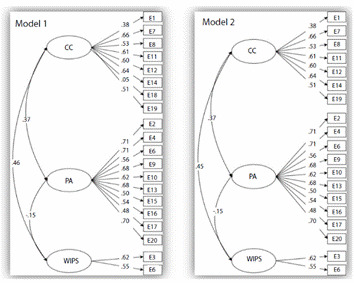
Variations in factor loadings and inter-factor correlations

*Note: Model 1 = 20 items; Model 2 = 19 items (item 18 left out); CC = Compassionate care; PT = Perspective-taking; SPS/WIPS = Standing in the patient's shoes*

Regarding reliability, the Omega coefficient yielded indices of 0.73, 0.86, and 0.51 for the CC, PT and SPS dimensions, respectively, in the model that included item 18. In the revised model, the indices were 0.76, 0.86, and 0.51. It is worth mentioning that invariance by sex could not be demonstrated due to the great heterogeneity within both groups by sex and program. Figure 1 shows the path diagrams for the 20-item model (1) and the revised model leaving out item 18 (2), indicating that variations in factor loadings and inter-factor correlations were virtually nonexistent.

Table 3 presents the estimates of mean, standard deviation, and sample size for empathy and its dimensions. Medical students scored higher than nursing students in empathy and each one of its dimensions. The same occurred in the distribution by sex, with one exception in the SPS dimension, where female nursing students scored higher than their medical counterparts. Female students generally showed higher empathy levels across all dimensions than male students.

**Table 3:** Results of the estimation of descriptive statistics for empathy and its dimensions

		Medicine (4g36)			Nursing (237)	
	Male (122)		Female (314)	Male (49)		Female (188)
Empathy or total scale		103.14 ± 71.33			91.50 ± 68.80	
	99.04 ± 71.50		104.76 ± 70.04	89.28 ± 59.59		92.06 ± 70.92
CC dimension		37.63 ± 45.26			35.27 ± 34.76	
	35.05 ± 49.32		38.65 ± 42.64	32.37 ± 36.41		36.00 ± 33.61
PT dimension		57.60 ± 41.81			47.95 ± 53.91	
	56.20 ± 42.48		58.15 ± 41.35	49.49 ± 48.46		47.56 ± 55.14
SPS dimension		7.91 ± 12.13			8.28 ± 13.41	
	7.79 ± 13.03		7.97 ± 11.77	7.49 ± 13.66		8.50 ± 13.20

*CC = Compassionate care; PT = Perspective-taking; SPS = Standing in the patient's shoes; n = Sample size.*

Table 4 shows the results of the two-way ANOVA, effect size, and test power. For overall empathy, differences were found between programs and sex. Medical students had higher empathy values than nursing students, and female students showed higher empathy levels than male students. The same pattern was observed for CC dimension. However, in the PT dimension, differences were found only between programs, with medical students showing higher levels than nursing students. For SPS dimension, no differences were observed between programs, but sex differences were noted, as women performed better than men. In all cases where differences were found, effect sizes varied, ranging from small (around 0.01) to moderate (around 0.06). The power of the test exceeded 0.80 in all cases, except for the differences found by sex in the SPS dimension.

In general terms, significances observed for program and sex involved small to moderate differences, and the power of the test indicates that these variations were representative of the differences within the student populations evaluated. The estimated R2 values were 0.126 for empathy, 0.0390 for CC, 0.169 for PT, and 0.009 for SPS.

**Table 4:** Results of the two-way ANOVA, effect size, and power of the test

	Factors	F	^p^	ἠ2	PP
E	Program	56.02	0.001	0.08	1.00
	Sex	8.03	0.005	0.01	0.81
	Program * Sex	0.96	0.329	0.01	0.16
CC	Program	9.06	0.003	0.01	0.85
	Sex	16.64	0.0001	0.02	0.98
	Program * Sex	0.001	0.981	0.001	0.05
PT	Program	75.48	0.001	0.10	1.00
	Sex	0.001	0.995	0.001	0.05
	Program * Sex	3.80	0.052	0.01	0.49
SPS	Program	0.10	0.758	0.001	0.06
	Sex	5.47	0.020	0.01	0.65
	Program * Sex	2.81	0.094	0.01	0.39

*E = Empathy; CC = Compassionate care; PT = Perspective-taking; SPS = Standing in the patient's shoes; n = Sample size. tf* ^*2*^ = Eta squared; PP = Power of the test. * = Interaction between the factors studied.

## Discussion

The results of the psychometric analysis on empathy data from nursing and medical students showed that the model fitted when appropriate techniques were integrated into the psychometric analysis and model fit was demonstrated. The importance of a proper fit of the theoretical model of an instrument adds robustness to the study conclusions, while calculating values without ensuring model fit may lead to biases in the estimates of empathy construct levels, which may induce uncontrolled errors^13^^, ^^33^. Therefore, several authors have consistently emphasized the importance of routine psychometric analysis in empathy studies to ensure that the model fits the data under assessment^1^^, ^^2^^, ^^6^^, ^^16^^, ^^17^^, ^^19^ and that empathy level estimates are free from biases caused by an under fitted model. One of the characteristics of empathy studies in Latin America is the presence of various cultural models across different countries. Culture may affect responses to instrument items and change the internal structure of the original item composition within each dimension. Of course, this would imply a quantitative distortion of the theoretical values in each dimension of empathy^34^^, ^^35^.

The duration of nursing and medicine programs can influence the development of student empathy in a number of ways. Longer programs, such as medicine (12 semesters compared to 8 semesters for nursing) may provide students with more time for clinical exposure, personal development, and emotional maturity, which could contribute to a greater capacity for empathy. Moreover, the extended duration could allow for a greater variety of clinical and learning experiences, further expanding students' perspectives. However, other factors such as the curriculum design and educational environment also play important roles in empathy development^36^^, ^^39^.

Some nursing students may choose this major due to the financial limitations to access to medicine programs, which can also impact nursing students’ empathy compared to medicine students. This situation may cause feelings of frustration or resignation, potentially affecting their emotional commitment and identification with the nursing profession. As a result, some students may not feel the same commitment or emotional connection to nursing as they might to medicine, which would translate into a lower perceived empathy within their major. However, it is worth highlighting that this is not true for all nursing students, as many may develop strong empathy and commitment to their profession through their academic and clinical experiences^39^^, ^^40^.

Medicine students are exposed to various specialties throughout their education, which can significantly influence their empathy. They have the opportunity to experience a wide range of clinical situations and interact with patients who are facing different health challenges. Such interactions may foster a deeper understanding of patients' needs and concerns, potentially contributing to higher empathy levels in medical students compared to those in nursing programs^40^.

The way nursing profession is approached, focusing on direct patient care, while medical school tends to cover a broader scope of medical knowledge and responsibilities, can negatively influence perceptions of empathy toward the nursing major^41^. This is because medical care is often associated with broader roles involving diagnostic and therapeutic decisions, whereas nursing centers on more practical, hands-on aspects of direct patient care^42^.

In terms of sex, empathy (and its dimensions) in Latin American medicine and nursing programs shows variability. At times, male population in Latin America is sometimes found more empathetic than the female population. However, at other times, the inverse is found, while sometimes no sex differences are noted at all^1^^, ^^6^^, ^^9^. These findings cast doubt on whether women are necessarily more empathetic than men and suggests that empathy is the product of several factors such as culture, social and neurobiological modeling, early socialization, structural and functional brain variations, and genetic and hormonal factors^43^.

The results observed in the levels of empathy and its dimensions by program can be classified using the cut-off points estimated for nursing and medicine students in Latin America. For nursing students, the values observed for empathy (E), CC, PT, and SPS were 91.5, 35.27, 47.95, and 8.28, respectively (Table 3). Comparing these with the estimated cut-off points, E was high but at the lower limit of the 5th percentile (P5); CC had medium values (P50); PT was high (P25); and SPS was medium (P75). For medicine students, the values obtained were E=103.4, CC=37.63, PT=57.50, and SPS=7.91 (Table 3). When compared with the cut-off points, E was classified as medium (P90), CC as high (P25), PT as high (P50), and SPS as medium (P90). These findings show that nursing students face challenges in understanding the patients' emotions and in their ability to assist others, which can impact patient care.

Additionally, they face challenges in understanding the subjectivity of patients' thinking. In the case of medical students, their difficulties in grasping the subjectivity of the patients' thoughts were apparent, as they evidently found it difficult to understand what patients think and how they feel about their conditions, even though other dimensions were rated as high (but below the P50). This situation may explain the medium classification for overall empathy (E).

Despite the high percentage of the sample compared to the population size, the data of students who did not answer the instrument may entail some biases that prevent us from reaching more accurate conclusions about this population, which can be considered a limitation to this study. However, in spite of the above, the conclusions represent consistent trends that justify a well-targeted empathy intervention to overcome specific empathy deficiencies and strengthen the dimensions in which students perform well. Nonetheless, there is a need to study whether empathy is influenced by other factors such as age, religion, previous working experience as nursing assistants, economic stratum, adverse events during internships or during the major, or previous training courses. Furthermore, it is also necessary to explore potential modulating variables of empathy (and their dimensions) such as individual resilience, family functioning, personality, stress, and academic motivation, among other variables, using structural equations.

## Conclusion

Based on the findings, it is possible to diagnose the empathy levels of students of both programs studied. For the nursing student, although their overall empathy scores are “high”, they have deficiencies in the CC and SPS dimensions with the previously discussed consequences. Medical students, on the other hand, show deficiencies in the SPS dimension, which may explain their medium empathy scores. From these data, it is possible to design an intervention aimed at increasing the levels of the deficient empathy dimensions in nursing and medical students.
